# Cold acclimation has a differential effect on leaf vascular bundle structure and carbon export rates in natural *Arabidopsis* accessions originating from southern and northern Europe

**DOI:** 10.1002/pld3.251

**Published:** 2020-08-10

**Authors:** Katja Schneider, Lorena Abazaj, Cornelia Niemann, Laura Schröder, Thomas Nägele

**Affiliations:** ^1^ Department Biology I Plant Development LMU München Planegg‐Martinsried Germany; ^2^ Department Biology I Plant Evolutionary Cell Biology LMU München Planegg‐Martinsried Germany

**Keywords:** *Arabidopsis thaliana*, cold acclimation, natural variation, photosynthesis, source‐sink interactions, vascular bundle

## Abstract

Acclimation to low but non‐freezing temperature represents an ecologically important process for *Arabidopsis thaliana* but also for many other plant species from temperate regions. Cold acclimation comprises and affects numerous molecular and physiological processes and the maintenance of sugar supply of sink tissue by photosynthetically active source tissue is essential for plant survival. Here, changes in vascular bundle (VB) structure at the leaf petiole were analysed together with sucrose exudation rates before and after cold acclimation. Six natural *Arabidopsis* accessions originating from southern and northern Europe were compared. Photosynthetic efficiency, that is, maximum and effective quantum yield of photosystem II, revealed a significant effect of environmental condition. Only for northern accessions was a highly significant negative correlation observed between leaf sucrose exudation rates, xylem, and petiole cross‐sectional areas. Furthermore, only for northern accessions was a significant increase of VB and leaf petiole cross‐sectional area observed during cold acclimation. In contrast, variance of cross‐sectional areas of cold acclimated southern accessions was strongly reduced compared to control plants, while mean areas remained similar under both conditions. In summary, these findings suggest that natural *Arabidopsis* accessions from northern Europe significantly adjust sink strength and leaf VB structure to maintain plant growth and photosynthesis under low temperature.

## 1 INTRODUCTION

The exposure of higher plants to low but non‐freezing temperature induces a tightly regulated, multigenic and multifaceted process termed cold acclimation. Cold acclimation is a crucial process for many plant species of the temperate zone because low temperature significantly impacts the geographic range over which they occur (Hoffmann, 2002). It comprises and affects gene expression, translational, and post‐translational processes and induces significant alteration in metabolic pathway regulation (Wang et al., 2017, Bahrani et al., 2019, Liu et al., 2019). To sustain a stable metabolic homeostasis during cold exposure, photosynthetic capacities and metabolism of carbohydrates need to be tightly adjusted to prevent toxic accumulation of reactive oxygen species (ROS) (Dreyer and Dietz, 2018). Although it is known that plants alter the composition and activation state of their photosynthetic apparatus due to environmental changes within a process termed photosynthetic acclimation, many of the involved molecular mechanisms are still not understood. Regulation of metabolic pathways, and particularly of sucrose (Suc) metabolism, are well known to play a central role in photosynthetic acclimation (Strand et al., 2003, Stitt et al., 2010, Nägele et al., 2012, Herrmann et al., 2019). In the light, photosynthetic CO_2_ fixation results in triose phosphates that are substrate for starch biosynthesis in the chloroplast and Suc biosynthesis in the cytosol that is regulated by cytosolic fructose‐1,6‐bisphosphatase (cFBPase) and sucrose phosphate synthase (SPS) (Strand et al., 2000). Suc represents a central transport sugar in many plant species; it plays an important role as storage compound, represents an osmotically active solute and is involved in sugar signaling (Ruan, 2014). Recent findings suggest that Suc export from the chloroplast into the cytosol is involved in cold acclimation and is required for development of maximal freezing tolerance (Patzke et al., 2019). The authors discussed that Suc in the chloroplast serves as a reservoir to supply cytosolic and vacuolar sugar metabolism under stress conditions. Our own studies indicated that Suc compartmentation and invertase‐driven cleavage in the cytosol and vacuole significantly affect the cold stress response and stabilization of photosynthesis (Nägele and Heyer, 2013, Weiszmann et al., 2018).

Stabilization of Suc supply for carbon sink organs, for example, roots, by photosynthetically active source tissue is essential for plant growth and development under changing environmental conditions. For transport, Suc is loaded into the phloem that ultimately establishes a source‐sink hydrostatic pressure differential that drives phloem transport. In the sinks, Suc is unloaded and provided for cellular metabolism. Readers interested in details of phloem loading, transport and unloading, are asked to refer to more detailed and specialized literature at this point (e.g., Johannes and Patrick, 2017). In general, Suc is secreted into the cell wall space by passive SUGARS WILL EVENTUALLY BE EXPORTED TRANSPORTERs (SWEETs) and loaded into the phloem by SUCROSE TRANSPORTERs (SUCs or SUTs) (Braun, 2012). A recent study demonstrated that sucrose transporter SUC2, which regulates carbon export from leaves of *Arabidopsis thaliana*, is controlled via ubiquitination and phosphorylation that directly links whole plant carbon homeostasis to posttranslational modification (Xu et al., 2020). Furthermore, previous studies have shown that regulation of SUTs is part of an abiotic stress response (see e.g., Gong et al., 2015) and that both transcriptional and posttranscriptional mechanisms are involved in regulation of Suc export (Xu et al., 2018).

Due to the availability of natural accessions with a broad longitudinal and latitudinal range of geographical origin, *Arabidopsis thaliana* has become an attractive system to study plant‐environment interactions, ecological, and evolutionary patterns (Bouchabke et al., 2008, Mishra et al., 2014, Mönchgesang et al., 2016, The 1001 Genomes Consortium, 2016, de Jong et al., 2019). Cold acclimation capacity differs significantly between natural *Arabidopsis* accessions, and accessions with differential cold and freezing tolerance significantly differ in regulation of photosynthesis and carbohydrate metabolism (Hannah et al., 2006, Fürtauer et al., 2019, Zuther et al., 2019). Yet, many aspects still remain unclear about the stabilization of carbohydrate homeostasis under low temperature. Particularly, natural variation of regulatory and developmental processes involved in the stabilization of source‐sink interactions remain elusive. Comparing foliar vasculature, photosynthesis and transpiration rates in three natural *Arabidopsis* accessions revealed that photosynthetic capacity and foliar phloem features varied consistently with latitude and temperature of the habitat of origin (Adams et al., 2016). Vein density and water‐transporting tracheary elements were found to vary with precipitation level of the habitat (Adams et al., 2016). These findings provide clear evidence that, in addition to the molecular level, for example, the regulation of SUC2, structural and anatomical properties also need to be addressed (Davidson et al., 2011).

In the present study, we analysed natural variation of leaf Suc exudation and vascular bundle (VB) structure at leaf petioles during cold acclimation. We compared cross‐sectional areas of VB tissue and recorded leaf Suc exudation rates under 22°C and 4°C in six natural accessions originating from southern and northern Europe. Additionally, we recorded chlorophyll fluorescence parameters to monitor photosystem II efficiency before and after cold acclimation.

## 2 MATERIALS AND METHODS

### 2.1 Plant material and growth conditions

Plants of natural *Arabidopsis thaliana* accessions Ct‐1 (southern accession, Italy; longitude E15.0, latitude N37.3; altitude 1‐100m), C24 (southern accession, Portugal; longitude W8.42, latitude N40.21; altitude ‐), Fei‐0 (southern accession, Portugal; longitude W8.54, latitude N40.92; altitude 140 m), Col‐0 (northern accession, Poland; E 15.7 N52.7, based on information given in El‐Lithy et al., 2004; altitude ‐), Rsch‐4 (northern accession, Russia; longitude E34.0, latitude N56.3; altitude 100‐200 m), and Oy‐0 (northern accession, Norway; longitude E6.19, latitude N60.39; altitude 1–100 m) used in this study were grown on a 1:1 mixture of GS90 soil and vermiculite in a climate chamber under short‐day conditions (8 hr/16 hr light/dark; 90 µmol photons m^−2^ s^−1^; 22°C/16°C; 70% relative humidity). Seeds were obtained from the Nottingham *Arabidopsis* Stock Centre (NASC). Four plants of each accession were grown in a pot of 729 cm^3^ volume. After 4 weeks, plants were transferred to the greenhouse and grown under long day conditions (16 hr/8 hr light/dark, 100 µmol photons m^−2^ s^−1^). After 7 days in the greenhouse and after 6–9 hr in the light, plants were either (a) sampled (microscopy, exudation) and used for chlorophyll fluorescence measurements (“control”), or (b) transferred to a cold room for temperature acclimation (4°C, 16 hr/8 hr light/dark; 90 µmol photons m^−2^ s^−1^). After 7 days at 4°C, cold acclimated plants were sampled again after 6–9 hr in the light (microscopy, exudation) and used for chlorophyll fluorescence measurements. For analyses, mature leaves that had expanded fully before cold acclimation were used.

### 2.2 Chlorophyll fluorescence measurements

The maximum quantum yield of PSII (Fv/Fm) was determined after 15 min of dark adaptation by supplying a saturating light pulse. Dynamics of quantum efficiency of PSII (ΦPSII) and photochemical quenching (qP) were determined within light response curves by stepwise increase of photosynthetically active radiation (PAR) from 0 to 1,200 µmol photons m^−2^ s^−1^ in 5 min intervals. All measurements were performed using a WALZ^®^ GFS‐3000FL system equipped with measurement head 3010‐S and *Arabidopsis* chamber 3010‐A (Heinz Walz GmbH, https://www.walz.com/). Cold exposed plants were acclimated to ambient temperature, that is, 22°C, for 20 min before measurement.

### 2.3 Sample preparation, staining and semi thin sectioning for light microscopic analysis

Petioles of *Arabidopsis* leaves were cut into 1 mm pieces in buffer (75 mM cacodylate, 2 mM MgCl_2_, pH 7.0) and stained over night at room temperature in a 1:1 mixture of safranin/astra blue (1% (w/v) safranin in ddH_2_O/ 0.1% (w/v) astra blue, 2% (w/v) tartaric acid). Samples were dehydrated with ethanol (2x 10 min 50% (v/v) EtOH; 3x 100% EtOH for 10, 30, and 60 min) and embedded in Spurr´s resin. For each genotype under both analysed conditions, at least 25 cuts from at least five different plants were prepared. For preparation of semi thin sections, five samples of each “genotype × condition pool” were randomly chosen for further preparation. Semi thin sections (thickness, 2 µm) were cut using a diamond knife on a Reichert Ultracut‐E ultra‐microtome. Sections were mounted on glass slides and examined using a Zeiss Axiophot light microscope. Images were acquired using a SPOT insight 2 MP CCD color digital camera. Light microscopic images were used to measure the areas of total petiole, VBs and xylem. Area of other VB tissue than xylem was calculated as the difference of total VB area and xylem area. Examples for images and area definitions of petiole and VB tissues are provided in the supplements (Figures S1 and S2).

### 2.4 Sucrose quantification from exudates

To determine the sucrose concentration from exudates rosettes from plants grown under control conditions and after cold acclimation were placed in a petri dish containing 20 mM EDTA solution (pH 8.0). Leaves were cut with a razor blade at the centre of the rosettes. Five leaves from each plant were pooled and placed in a 1.5 ml reaction tube filled with 20 mM EDTA (pH 8.0) and exposed to growth PAR intensity, that is, 90 µmol photons m^−2^ s^−1^, for 1 hr. This was repeated five times independently for each accession under each condition. Exudation was stopped by removing the leaves from the tube. Leaves were carefully dried on a paper towel and weighed on a fine scale to determine the fresh weight per exudate. The entire exudate was dried in a desiccator. Exudation experiments were performed at 22°C with control plants and at 4°C with cold acclimated plants. Dried exudates were resolved in 300 µl ddH_2_O. 100 µl of solvent containing exudate from the leaf was mixed with 100 µl 30% (w/v) KOH and boiled at 95°C for 10 min. After quick cooling on ice 1 ml of anthrone reagent (0.14% (w/v) anthrone in 14.6 M sulfuric acid) was added, briefly mixed by inverting and incubated at 40°C for 30 min. Extinction was measured immediately at 620 nm using a microplate reader (Synergy H1, BioTek, www.biotek.com).

### 2.5 Statistics and area measurements

For statistical data evaluation, we used the free software environment R Version 3.6.1 (https://www.r‐project.org/) (R Core Team, 2019) and RStudio Version 1.2.5019 (https://rstudio.com/) (RStudio Team, 2019). Area measurements were done in ImageJ 1.52A using the “Area” tool (https://imagej.nih.gov/ij/index.html).

## 3 RESULTS

### 3.1 Geographical origin has no significant effect on the photosynthetic efficiency of cold acclimated plants

**FIGURE 1:**
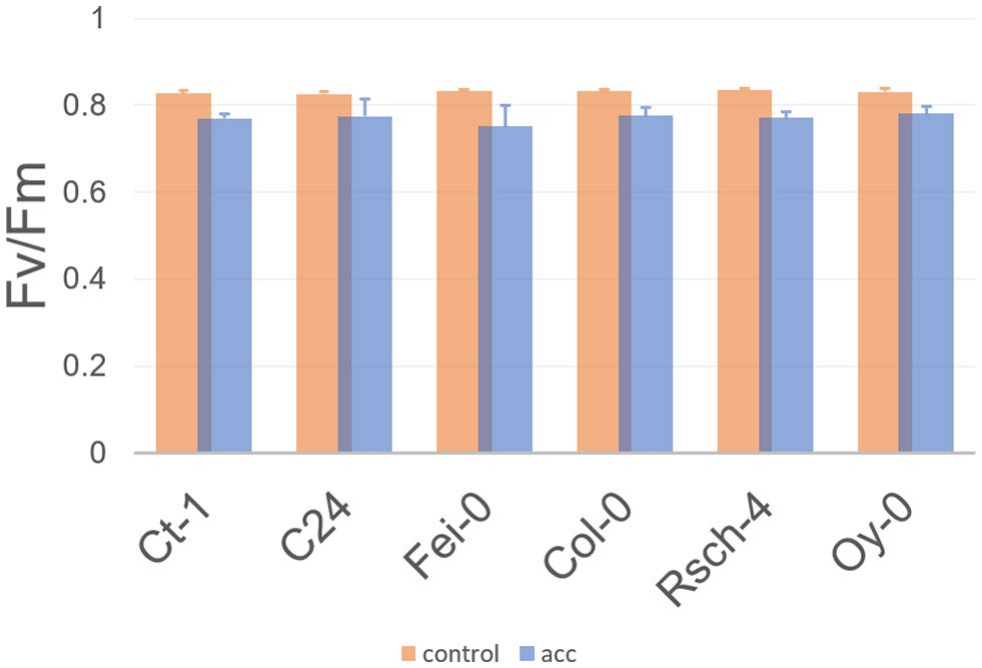
Maximum quantum yield of PSII in accessions before and after cold acclimation. Bars represent mean values ± SD (*n* = 3). Orange bars: control plants; blue bars: cold acclimated plants (7 days at 4°C)

In all accessions, maximum quantum yield of PSII (Fv/Fm) significantly dropped from 0.82 to 0.83 under control conditions to 0.77–0.78 after cold acclimation (Figure 1; ANOVA, *p* < .05). In accession Fei‐0, Fv/Fm dropped to the lowest mean level (0.75) which was, however, not significantly lower than in other accessions. Fv/Fm was recorded following 15 min of leaf dark adaptation. Following Fv/Fm measurement, light response curves were recorded to determine dynamics of quantum efficiency of PSII (ΦPSII) and qP under different PAR intensities. Similar to Fv/Fm, ΦPSII and qP were significantly reduced in cold acclimated plants compared to control plants across all accessions (ANOVA, *p* < .001), while no significant “genotype effect” was observed under light intensity of 90 µmol photons m^−2^ s^−1^. For both parameters, the difference between mean values of control and cold acclimated plants increased with PAR intensity applied within the light response curves (Figures S3 and S4). In summary, cold acclimation had a significant effect on all quantified photosynthetic parameters while no significant effect was found to differentiate accessions from southern and northern Europe.

### 3.2 The reduction of leaf exudation rates of sucrose during cold acclimation is more distinct in accessions from northern than from southern Europe

**FIGURE 2:**
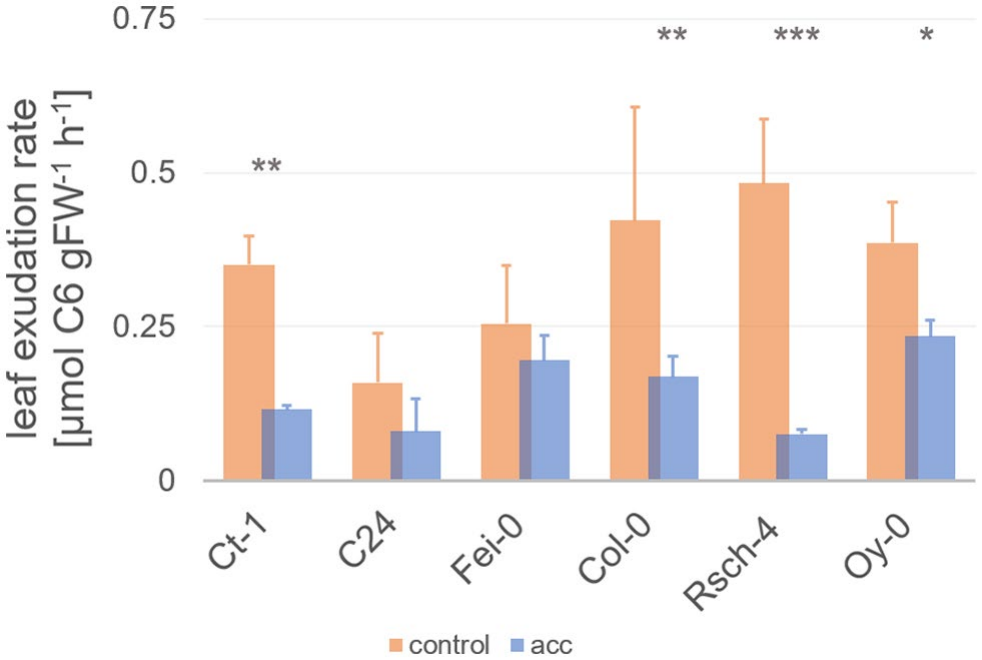
Leaf exudation rates before and after cold acclimation. Bars represent mean values ± SD (*n* = 5). Orange bars: control plants; blue bars: cold acclimated plants (7 days at 4°C). Asterisks indicate significance (ANOVA). *p < .05; **p < .01; ***p < .001

The stabilization of carbon supply to non‐photosynthetically active sink organs, for example, roots, by source leaves is essential for successful acclimation to low temperature. We quantified the exudation rate of sucrose for control plants and cold acclimated plants under each respective growth temperature, that is, at 22°C for control plants and at 4°C for cold acclimated plants. Under control conditions, northern accessions had a significantly higher leaf exudation rate than southern accessions (Figure 2; ANOVA, *p* < .001). In contrast, after 7 days of cold acclimation, no significant difference between exudation rates quantified at 4°C was observed between southern and northern accessions. Remarkably, exudation rates in two out of three southern accessions, C24 and Fei‐0, dropped only slightly and not significantly at 4°C. Exudation rates in Ct‐1 and all northern accessions decreased significantly during cold acclimation (ANOVA, *p* < .05). The most distinct reduction of exudation rates was observed in the Russian accession Rsch‐4 from ~0.5 to 0.07 µmol C6 gFW^−1^ hr^−1^.

### 3.3 Cold acclimation induces structural changes in VB structure at the leaf petiole

**FIGURE 3:**
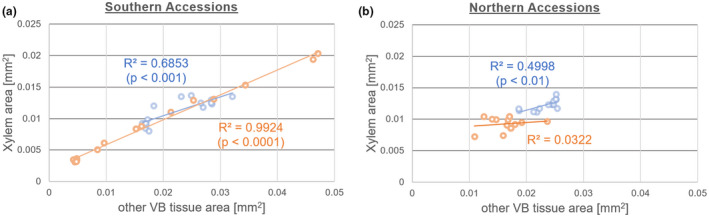
Correlation of xylem and other VB tissue area at leaf petioles. (a) Areas derived from microscopy of southern accessions Ct‐1, C24, and Fei‐0. (b) Areas derived from microscopy of northern accessions Col‐0, Rsch‐4, and Oy‐0. Orange colored: control samples (*n* = 4–5); blue colored: cold acclimated samples (*n* = 4–5). Significance is indicated by *p*‐values of Pearson correlations. An interactive accession‐specific graphical representation is provided in the supplements (Figures S5 and S6). Cross‐sectional areas are provided in Table S1. VB, vascular bundle

Evaluation of xylem and other VB tissue areas from light microscopic images of leaf petiole cross sections revealed a positive correlation across all accessions and conditions (Figure 3). Under control conditions, xylem and other VB areas were significantly correlated in southern accessions with *R* ^2^ = .9924 and *p* < .0001 (Pearson correlation). In contrast, only a weak positive (*R* ^2^ = .0322) and non‐significant correlation was observed in northern accessions. This changed during cold acclimation and resulted in a significantly positive correlation (*R* ^2^ = .4998, *p* < .01). Also, in cold acclimated plants of southern accessions such a significant and positive correlation was observed (*R* ^2^ = .6853, *p* < .001), which, however, compared to control conditions, decreased both in regression coefficient and significance.

### 3.4 Leaf exudation rate negatively correlates with xylem and petiole area in cold acclimated plants of northern accessions

**FIGURE 4:**
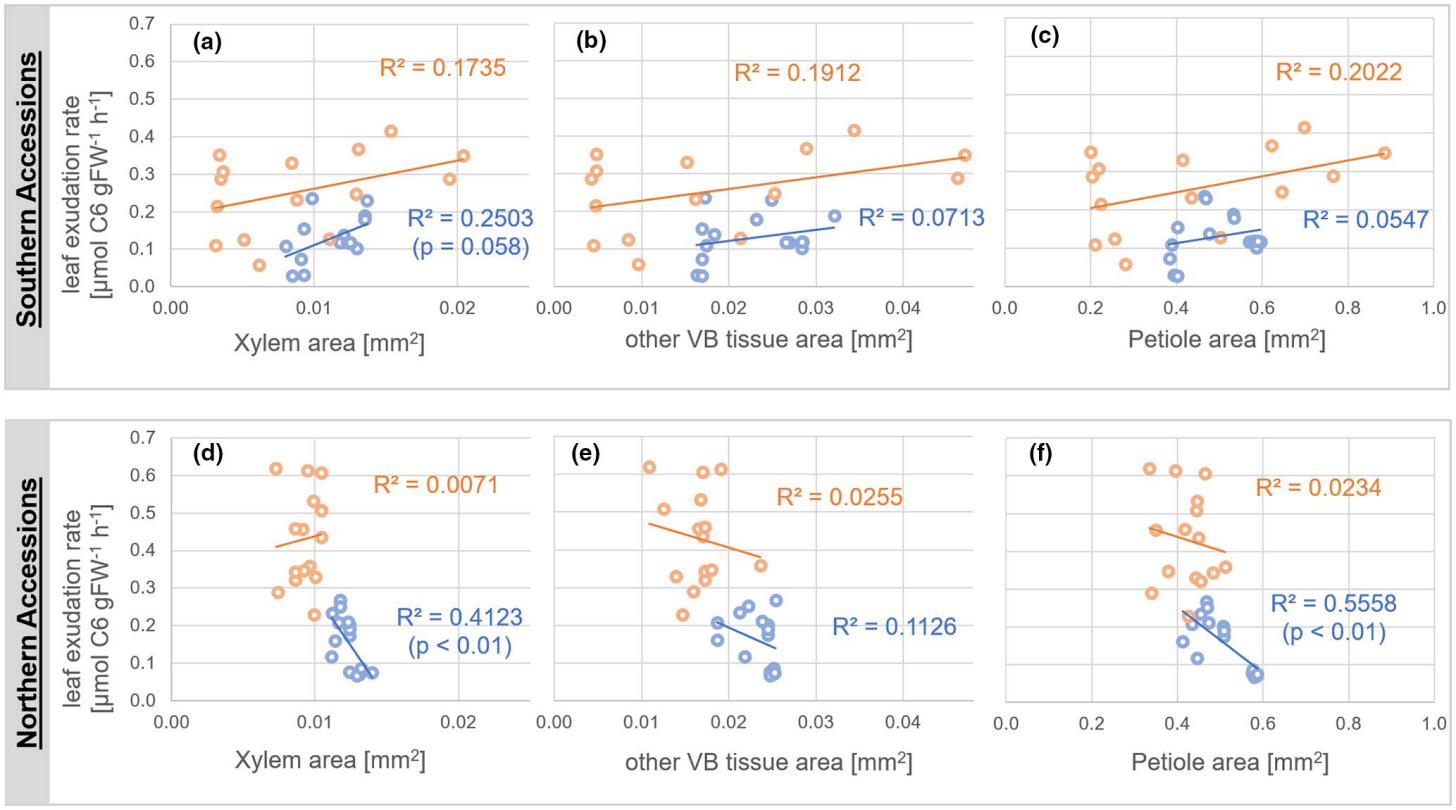
Leaf exudation rates as a function of cross‐sectional areas. Upper panel (a–c): leaf exudation rate as a function of (a) xylem area, (b) other VB tissue area, (c) petiole area of southern accessions Ct‐1, C24, and Fei‐0. Lower panel (d–f): leaf exudation rate as function of (d) xylem area, (e) other VB tissue area, (f) petiole area of northern accessions Col‐0, Rsch‐4 and Oy‐0. Orange colored: control samples; blue colored: cold acclimated samples (7 days, 4°C). Significance is indicated by p‐values of Pearson correlations. An interactive accession‐specific graphical representation is provided in the supplements (Figures [Link], [Link], [Link], [Link], [Link], [Link]). VB, vascular bundle

Leaf exudation rates were correlated with cross‐sectional areas of xylem, other VB tissue and the complete petiole (Figure 4). In the southern accessions Ct‐1, C24, and Fei‐0, cold acclimation induced a positive and nearly significant correlation of leaf exudation rate and xylem area (Figure 4A). Correlations with other VB tissue and petiole area remained similar to those observed under control conditions (Figure 4B,C). Interestingly, the variance of all areas in southern accessions became smaller due to cold acclimation. For example, xylem areas ranged between ~0.003 and 0.02 mm^2^ under control conditions but only between ~0.008 and 0.015 mm^2^ in cold acclimated plants (Figure 4A). A similar effect was observed for other VB tissue and petiole area (Figure 4B,C).

In northern accessions, that is, Col‐0, Rsch‐4, and Oy‐0, variance of all cross‐sectional areas remained similar before and after cold acclimation (Figure 4D–F). However, as already indicated in Figure 3, xylem areas significantly increased due to cold acclimation (*p* < .001, ANOVA) from a mean area of 0.009 mm^2^ before to 0.0123 mm^2^ after cold acclimation (increase ~32%). Other VB tissue and petiole areas also increased significantly by ~41% and ~20%, respectively.

In contrast to southern accessions, cold acclimation induced a significant and negative correlation of leaf exudation rates with xylem areas as well as with petiole areas in northern accessions (Figure 4D,F; *p* < .01). Although not significant, other VB tissue area was also negatively correlated to leaf exudation rates before and after cold acclimation. Thus, in summary, cold acclimation of northern accessions resulted in a pronounced and significant change of xylem areas that finally resulted in a negative correlation with leaf exudation rates.

### 3.5 Statistical integration of structural and physiological elements reveals significance of cold acclimation output in accessions from northern Europe

**FIGURE 5:**
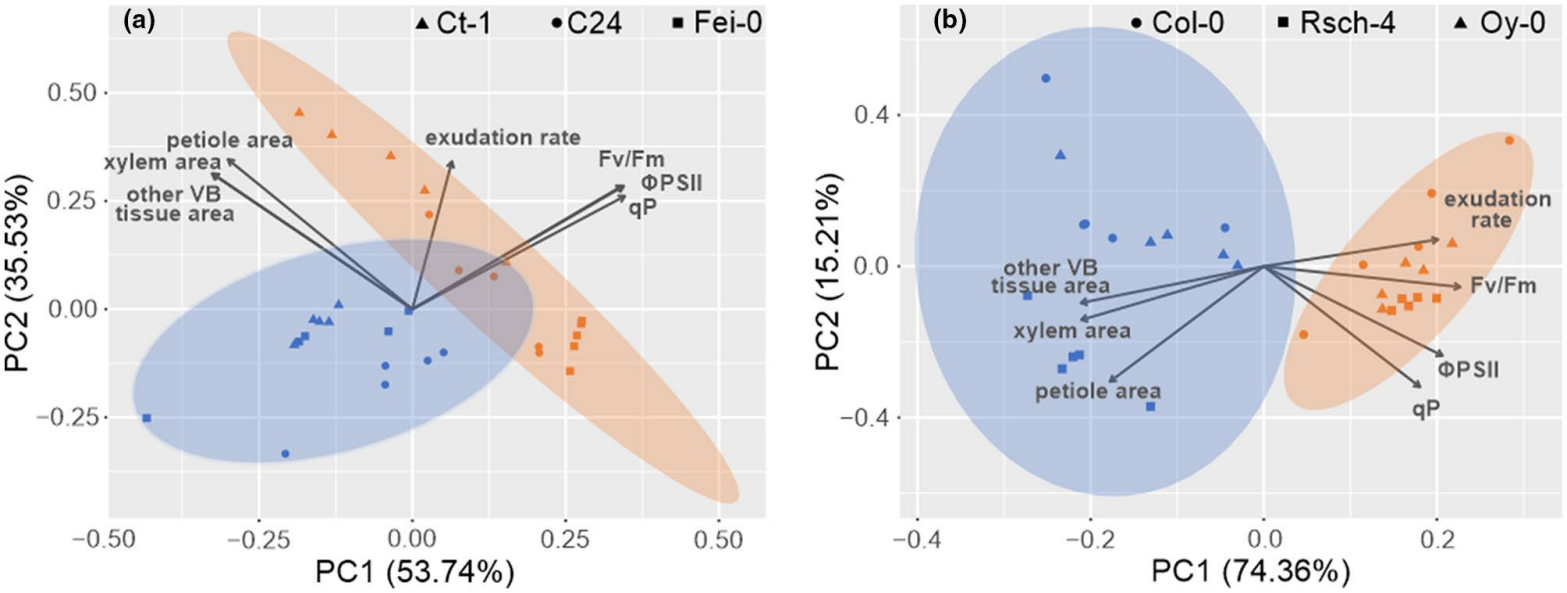
Principal component analysis of structural and physiological parameters. (a) PCA of southern accessions. Symbols represent independent samples of different genotypes: ▲Ct‐1, ●C24, ■Fei‐0. (b) PCA of northern accessions. Symbols represent independent samples of different genotypes: ●Col‐0, ■Rsch‐4, ▲Oy‐0. Orange colored: control samples; blue colored: cold acclimated samples (7 days 4°C). Arrows represent loadings. Confidence regions (95%) are indicated by colored ellipses. Data were scaled to zero mean and unit variance (autoscaling, z‐scores, *n* = 5). PCA, Principal component analysis

Principal component analysis (PCA) coupled with Hotelling’s T^2^ statistics revealed a significant separation of control and cold acclimated samples of northern accessions within a 95% confidence region, while, in contrast, confidence ellipses of southern accessions overlapped (Figure 5). PCA analysis comprised the variables Fv/Fm, ΦPSII, qP, exudation rate, petiole area, xylem area, and the area of other VB tissue, that is, the difference of VB area and xylem area. In both southern and northern accessions photosynthetic parameters, that is, Fv/Fm, ΦPSII, and qP, contributed similarly to the separation of both conditions. Yet, while in southern accessions the exudation rate factor loading was oriented in a nearly 90° angle toward xylem, other VB tissue and petiole areas (Figure 5A), the angle was almost 180° in northern accessions that strongly contributed to separation of both conditions along PC1 (Figure 5B). Among southern accessions, acclimation response differed between the Italian accession Ct‐1 and both Portuguese accessions C24 and Fei‐0. Control and cold acclimated samples of Ct‐1 were predominantly separated on PC2 that was determined by the significant effect on exudation rates (Figure 5A and Figure 2). In C24 and Fei‐0, control and cold acclimated samples were separated on PC1 that was, predominantly, determined by photosynthetic acclimation. Northern accessions displayed a more homogenous pattern and cold acclimation was found to be almost exclusively explained by PC1 that explained 20% more of the total variance than in southern accessions (54% vs. 74%; Figure 5B). In summary, northern accessions displayed a conserved cold acclimation response in which photosynthetic acclimation was consistently associated with acclimation of leaf exudation rates and increased cross‐sectional areas at the leaf petiole. In southern accessions cold acclimation response was more diverse and less significantly associated with quantified cross‐sectional areas at the leaf petiole.

## 4 DISCUSSION

Stabilizing carbohydrate supply of sink tissue by photosynthetically active source tissue is essential for plant acclimation to a changing environment. Particularly under low temperature, this stabilization is challenged by several factors. In source leaves, photosynthesis and carbohydrate metabolism need to be immediately adjusted if temperature drops to prevent an imbalance of photosynthetic primary and secondary reactions and irreversible cell and tissue damage. For example, zeaxanthin synthesis by de‐epoxidation of violaxanthin represents a fast mechanism of protective energy dissipation under chilling temperatures (Demmig‐Adams et al., 1989). Furthermore, cold acclimation typically comprises accumulation of osmolytes, increased proportion of unsaturated membrane lipids, increased leaf thickness which is, in apopolastic loaders, accompanied by a higher number of phloem cells per vein or, in symplastic loaders, by large minor vein phloem cells (Adams et al., 2018). Recorded data of the present study indicate a similar capability of accessions from southern and northern Europe to photosynthetically acclimate to 4°C. This finding is supported by previous studies that did not detect a correlation between classical chlorophyll fluorescence parameters, that is, Fv/Fm, ΦPSII or qP, and cold tolerance of natural *Arabidopsis* accessions at low but non‐freezing temperatures (Ehlert and Hincha, 2008, Distelbarth et al., 2013, Mishra et al., 2014). Furthermore, recorded light response curves indicated a similar capability of all accessions to cope with a dynamic range of PAR intensities before and after cold acclimation. Together with our previous studies, where we observed similar CO_2_ assimilation rates of cold acclimated plants of southern and northern accessions at 4°C (Nägele et al., 2011, Nägele et al., 2012, Nägele and Heyer, 2013), this provides further evidence for similar photosynthetic efficiency of all accessions under these conditions.

Discrimination of cold sensitive and tolerant accessions was shown to be possible using a different experimental and measurement design that applies combinatorial imaging together with the analysis of chlorophyll fluorescence transients of whole plant rosettes following mild sub‐zero temperature treatments (Mishra et al., 2014). However, the experimental setup chosen in the present study might have reduced accession‐specific differences of photosynthetic acclimation. For example, while in the present study plants were grown and acclimated under PAR intensities between 90 and 100 µmol photons m^−2^ s^−1^, previous work has shown differences in photosynthetic capacity between natural *Arabidopsis* accessions along a transect from Italy to Sweden when acclimated to low temperature under PAR intensity of 400 µmol photons m^−2^ s^−1^ (Adams et al., 2016). Growth at higher light intensities might reveal phenotypic differences between natural *Arabidopsis* accessions, but high PAR intensity may also result in photoinhibitory effects under low temperature (Murata et al., 2007) that might overlay strategies of metabolic regulation to stabilize sucrose export to sink tissue if sugar production exceeds utilization by the rest of the plant (Adams et al., 2013). Although only few accessions were analysed in the present study, sucrose exudation rates were found to contribute to discrimination of cold response in southern and northern accessions (see Figure 5). This indicates the physiological relevance of this variable for natural variation of cold acclimation which needs to be validated in future studies using more natural accessions. As indicated earlier, future studies might also combine growth and acclimation under low, moderate and high PAR intensities to provide further insight into plasticity of cold acclimation in natural *Arabidopsis* populations (Cohu et al., 2013).

Accessions from southern and northern Europe differed significantly in their VB structure that was estimated from transversal sections at the leaf petiole. As all leaves analysed here were already developed before cold exposure, data refer to neither leaves that developed at 4°C nor to young leaves in the centre of the leaf rosette. In northern accessions, cold acclimation induced a positive correlation between areas of xylem and other VB tissue, comprising also the phloem. In contrast, in southern accessions correlations were significant already under control conditions indicating less pronounced cold‐induced developmental effects on VB tissue. Remarkably, total variance of VB tissue areas was reduced during cold acclimation of southern accessions, which may suggest that VB tissue development is more constrained by low temperature than in northern accessions. Previous work has shown that leaf thickness together with sieve element cross‐sectional area per vein and loading cell number per vein positively correlates with the latitude of origin of natural *Arabidopsis* accessions growing at 12.5°C (with a resulting leaf temperature of 14°C; [Adams et al., 2016]). Yet, Adams and colleagues grew their plants under more than fourfold higher PAR intensity and used a bigger pot size to prevent potential growth limitation. This prevents a direct comparison of findings made by Adams and colleagues with findings of the present study but it still indicates a high plasticity of VB tissue development in *Arabidopsis* and a significant role for cold acclimation. Also, in context of light acclimation and phloem cell numbers of minor leaf veins, southern European accessions, here from Italy, were found to acclimate less pronounced than Swedish accessions (Adams et al., 2018). At 25°C and under PAR 1,000 µmol photons m^−2^ s^−1^, photosynthetic capacity together with leaf dry mass per area, number of palisade mesophyll cell layers and loading cell number per vein were found to correlate positively with the average annual habitat temperature of three accessions originating from Italy, Poland, and Sweden. Comparison with growth under 100 µmol photons m^−2^ s^−1^ revealed a weakened effect which reflects the dependency of effect size on PAR intensity (Adams et al., 2016, Stewart et al., 2017). Greater minor vein cross‐sectional sieve element areas found in high versus low light acclimated plants of Swedish accessions were hypothesized to be a prerequisite for their higher photosynthetic capacities (Cohu et al., 2013, Adams et al., 2018).

In the present and previous studies with growth light intensity significantly lower than 400 µmol photons m^−2^ s^−1^ and acclimation temperature of 4°C, no significant genotype‐effect of photosynthetic efficiency was observed that would discriminate between northern and southern accessions. In contrast, a less pronounced acclimation response was observed for vascular tissue of accessions originating from southern Europe compared to accessions from northern Europe. Remarkably, however, this lowered acclimation response did not result in a lowered rate of leaf sucrose exudation compared to northern accessions at 4°C (see Figure 2). Beyond that, two out of the three southern accessions rather seemed to stabilize exudation rates at 4°C to reach a value similar to that under control conditions, that is, 22°C. While it has been shown earlier that leaf exudation rates at low temperature did not significantly correlate with broad geographical origin of diverse plant species and phloem loadings types, that is, symplasmic and apoplasmic (Schrier et al., 2000), the observed significant and homogenous decrease of exudation rates in northern accessions still might indicate a cold acclimation strategy of *Arabidopsis thaliana*. It remains speculation at this point, but cold acclimation of northern accessions might prepare plants more efficiently for survival of sub‐zero temperatures and a significant reduction of sucrose export from leaves into sink tissue might support the accumulation of cryoprotectants and osmolytes (Zuther et al., 2018).

In contrast to southern accessions, correlations between leaf exudation rate, xylem, and petiole cross‐sectional areas in northern accessions became significantly negative during cold acclimation while xylem and petiole area increased (see Figure 4). The observed proportional increase of all monitored areas and a similar variance compared to control samples suggests that low temperature did not constrain leaf petiole growth to an extent observed for southern accessions that contributes to the phenotypic plasticity of *Arabidopsis* under low temperature (Atkin et al., 2006).

Although sucrose is transported in the phloem and xylem only indirectly contributes to leaf sucrose transport from source to sink tissue, the significance of the cold‐induced negative correlation of xylem and petiole areas with sucrose exudation rates might indicate a functional role in cold acclimation of northern accessions. Previously, we found that the northern cold tolerant accession Rsch has a higher growth rate in terms of shoot fresh weight accumulation during cold acclimation than the sensitive accession Cvi (Nagler et al., 2015). Thus, a higher growth rate might also be reflected by increased petiole and xylem areas of northern accessions in the present study. Finally, although it remains pure speculation at this point, maybe sink strength of northern accessions has been reduced during cold acclimation in order to facilitate carbon and energy supply for maintenance and biosynthesis of protective substances (Demmig‐Adams et al., 2018). In parallel to a higher shoot growth rate, this would explain the reduced exudation rates in cold acclimated plants and represent a central trade‐off for development under low temperature.

## AUTHOR CONTRIBUTIONS

KS, LA, CN, and LS performed experiments, KS and LA performed data analysis. TN analysed data and wrote the manuscript. All authors approved the manuscript.

## Supporting information

Fig S1

Fig S2

Fig S3

Fig S4

Fig S5

Fig S6

Fig S7

Fig S8

Fig S9

Fig S10

Fig S11

Fig S12

Table S1
