# Supplementary figures and images for: Cold acclimation has a differential effect on leaf vascular bundle structure and carbon export rates in natural Arabidopsis accessions originating from southern and northern Europe
Source: Plant Direct. 2020 Aug 10;4(8):e00251. doi: 10.1002/pld3.251 (PMC7416751; doi:10.1002/pld3.251)

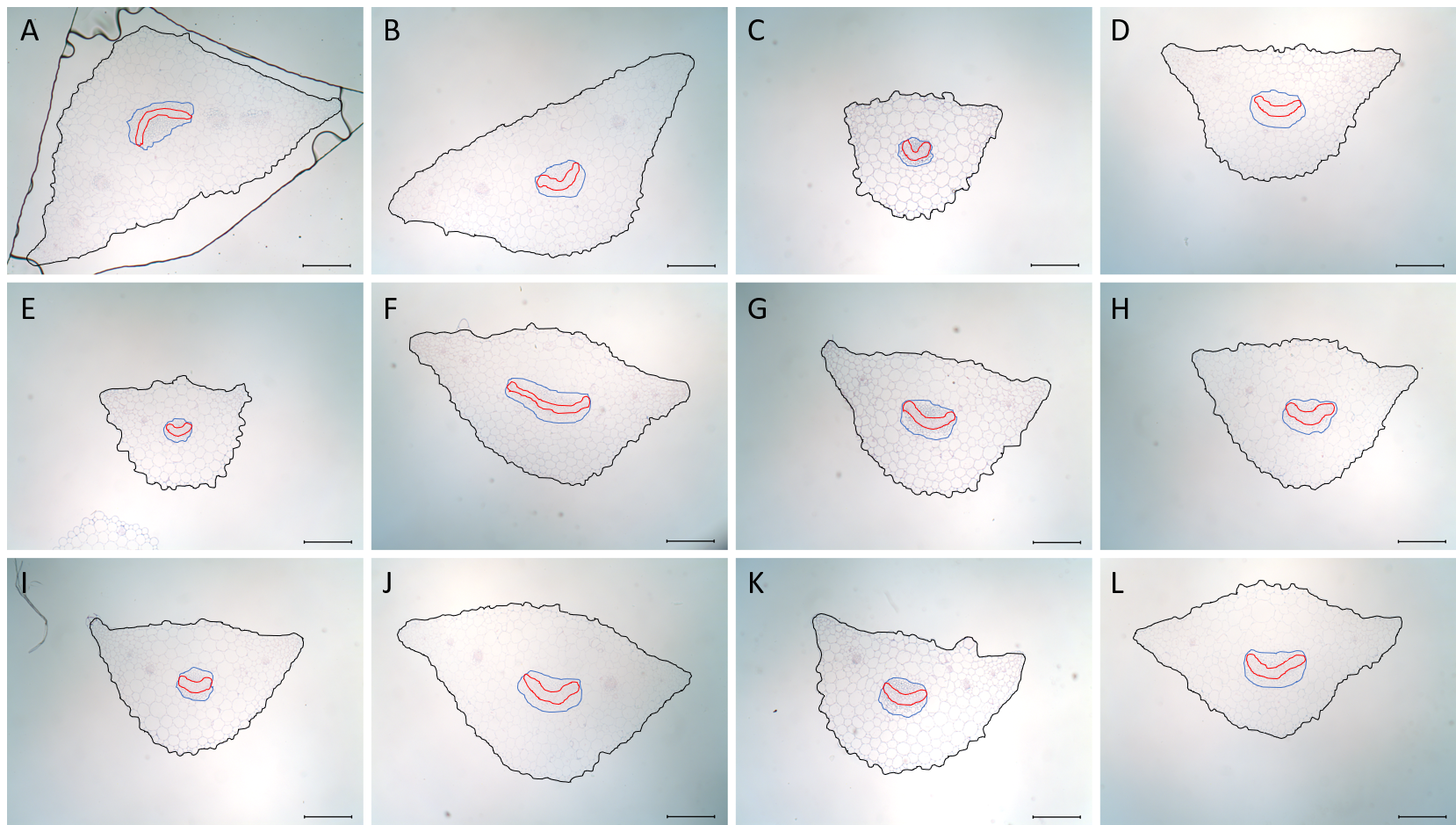

Supplement: Supplementary file 1 — Fig S1 [file PLD3-4-e00251-s001.tif]

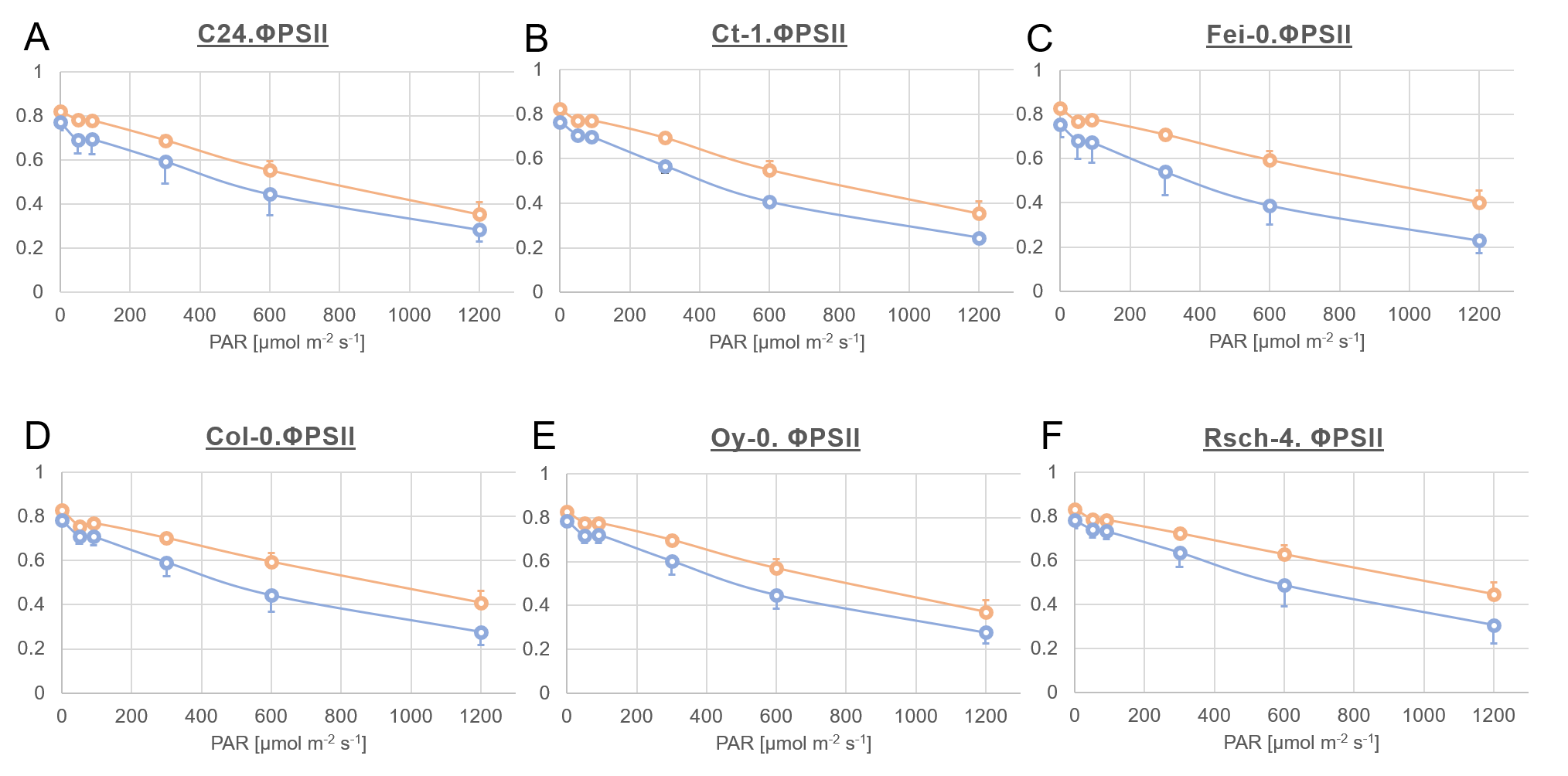

Supplement: Supplementary file 3 — Fig S3 [file PLD3-4-e00251-s003.tif]

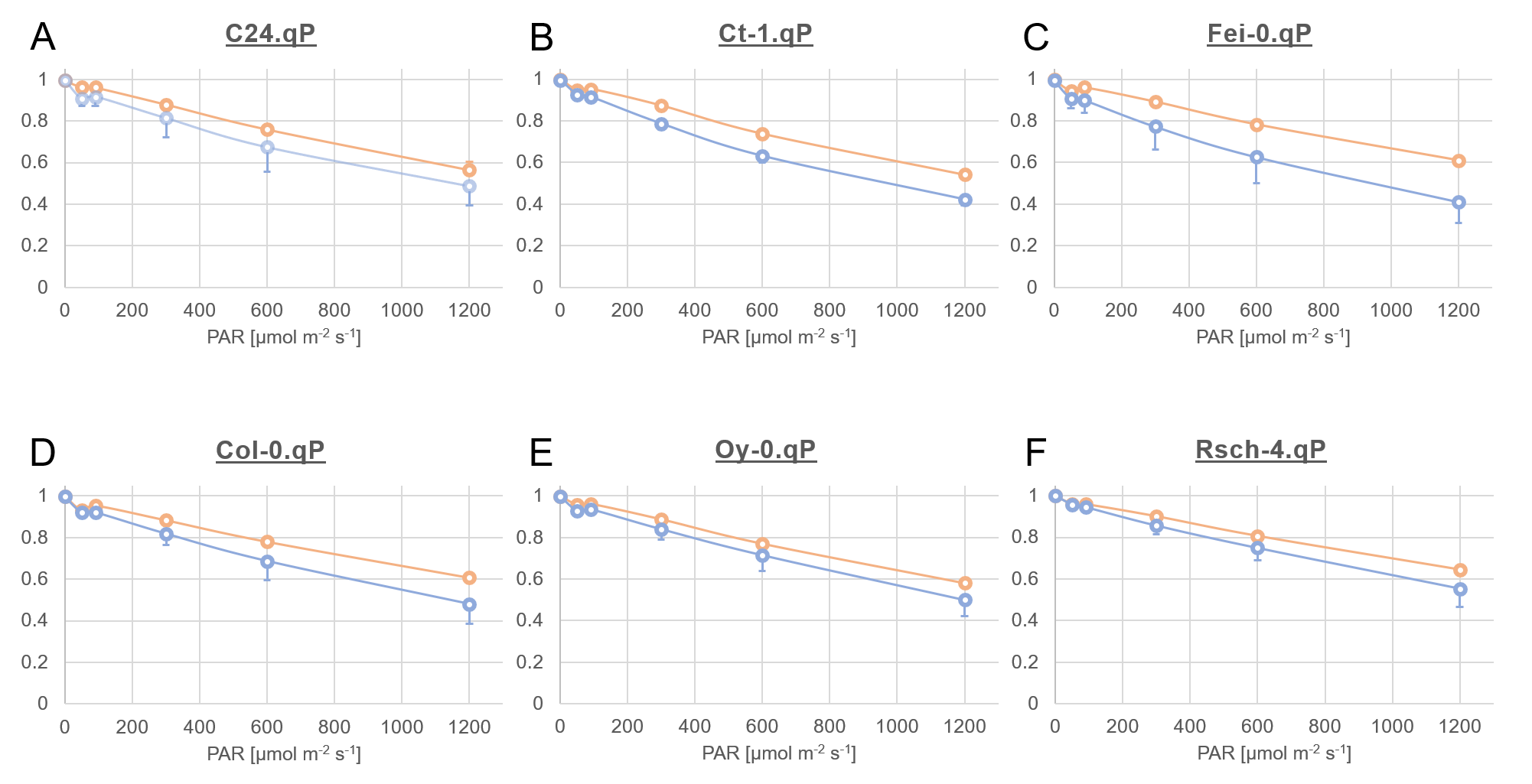

Supplement: Supplementary file 4 — Fig S4 [file PLD3-4-e00251-s004.tif]
