# Supplementary material for: Cold acclimation has a differential effect on leaf vascular bundle structure and carbon export rates in natural Arabidopsis accessions originating from southern and northern Europe
Source: Plant Direct. 2020 Aug 10;4(8):e00251. doi: 10.1002/pld3.251 (PMC7416751; doi:10.1002/pld3.251)
Supplement: Supplementary file 13 — Table S1 [file PLD3-4-e00251-s013.docx]

**Supplementary Table SI.** Cross-sectional areas at the leaf petiole (means ± SD, n=4-5).

| Accession | Condition | Xylem [mm^2^] | other VB tissue [mm^2^] | Petiole [mm^2^] |
| --- | --- | --- | --- | --- |
| Ct-1 | control | 0.01532 ± 0.0049 | 0.03434 ± 0.0132 | 0.67677 ± 0.1758 |
| Ct-1 | cold acclimated | 0.01242 ± 0.0004 | 0.02774 ± 0.0009 | 0.58444 ± 0.0102 |
| C24 | control | 0.00879 ± 0.0033 | 0.01616 ± 0.0073 | 0.42353 ± 0.1612 |
| C24 | cold acclimated | 0.00881 ± 0.0006 | 0.01691 ± 0.0004 | 0.39405 ± 0.0085 |
| Fei-0 | control | 0.00337 ± 0.0002 | 0.00461 ± 0.0002 | 0.21082 ± 0.0097 |
| Fei-0 | cold acclimated | 0.01253 ± 0.0016 | 0.02312 ± 0.0059 | 0.49505 ± 0.0354 |
| Col-0 | control | 0.00875 ± 0.0013 | 0.01684 ± 0.0048 | 0.40083 ± 0.0726 |
| Col-0 | cold acclimated | 0.01194 ± 0.0006 | 0.02278 ± 0.0026 | 0.47643 ± 0.0445 |
| Rsch-4 | control | 0.01023 ± 0.0003 | 0.01543 ± 0.0021 | 0.44890 ± 0.0089 |
| Rsch-4 | cold acclimated | 0.01313 ± 0.0006 | 0.02498 ± 0.0003 | 0.58060 ± 0.0055 |
| Oy-0 | control | 0.00882 ± 0.0003 | 0.01725 ± 0.0005 | 0.41600 ± 0.0540 |
| Oy-0 | cold acclimated | 0.01172 ± 0.0004 | 0.02226 ± 0.0026 | 0.45969 ± 0.0160 |
